# Targeted delivery of FAK siRNA by engineered exosomes to reverse cetuximab resistance via activating paraptosis in colon cancer

**DOI:** 10.1007/s10495-024-01986-x

**Published:** 2024-07-03

**Authors:** Yiting Geng, Wei Xia, Xiao Zheng, Lujun Chen, You Zhou, Jun Feng, Ye Yuan, Mingyue Zhang, Jianwen Lu, Shanshan Wei, Wenwei Hu

**Affiliations:** 1 Department of Oncology, The Third Affiliated Hospital of Soochow University, 185 Juqian Street, Changzhou, 213003 China; 2 Jiangsu Engineering Research Center for Tumor Immunotherapy, The Third Affiliated Hospital of Soochow University, Changzhou, 213003 China; 3 Tumor Biological Diagnosis and Treatment Center, the Third Affiliated Hospital of Soochow University, Changzhou, 213003 China; 4 Institute of Cell Therapy, Soochow University, Changzhou, 213003 China

**Keywords:** Colorectal cancer, Cetuximab resistance, Paraptosis, FAK, Engineered exosome

## Abstract

**Background:**

Cetuximab is extensively used in the treatment of metastatic colorectal cancer (mCRC). However, resistance poses a significant challenge to successful therapy. Recently, paraptosis, a non-classical programmed cell death, has garnered increased attention for its potential application value in antitumor treatments. We aimed to identify the essential pathways and signaling molecules involved in paraptosis inhibition and select them as therapeutic targets in cetuximab resistance. Additionally, engineered exosome technology is used as a drug delivery system with both targeted and effector properties.

**Results:**

By comparing the differential expression of paraptosis-related genes between drug-resistant colon cancer cells and sensitive cells, it was observed that the paraptosis level induced by cetuximab was significantly downregulated in drug-resistant cells. Kyoto Encyclopedia of Genes and Genomes (KEGG) analysis identified the focal adhesion kinase (FAK) signaling pathway as a key pathway involved in the suppression of paraptosis. The biological function of FAK in cetuximab-resistant cells was investigated through cell morphology observation, CCK-8 assay, colony formation assay, RT-qPCR, Western Blot, and loss-of-function experiments. The results showed that the FAK signaling pathway was significantly upregulated in cetuximab-resistant colon cancer cells, and siRNA interference targeting FAK could notably inhibit cell proliferation while upregulating the paraptosis level. Based on this, engineered colon cancer cells targeted and FAK siRNA loaded exosomes (CT-Exo-siFAK1) were constructed. In vitro experiments, CT-Exo-siFAK1 could effectively activate paraptosis and inhibit the proliferation of drug-resistant colon cancer cells. In vivo experiments also confirmed that CT-Exo-siFAK1 significantly suppressed tumor growth and metastasis while upregulating the paraptosis level.

**Conclusion:**

This study suggests that FAK signaling pathway-mediated inhibition of paraptosis levels is crucial in the sensitivity of cetuximab targeted therapy in colon cancer, and the use of engineered exosomes to deliver FAK siRNA may be an effective strategy to reverse cetuximab resistance.

**Supplementary Information:**

The online version contains supplementary material available at 10.1007/s10495-024-01986-x.

## Introduction

Colorectal cancer (CRC) frequently manifests insidiously, with roughly 15%-30% of patients presenting with metastases and 20%-50% of patients with initially localized cancer developing metastases [1]. Currently, the standard first-line treatment for RAS wild-type metastatic colorectal cancer (RASwt mCRC) involves the combination of anti-EGFR antibodies with chemotherapy but intrinsic and acquired resistance poses a significant challenge [2]. The overall response of tumors to treatment results from the cumulative effect of various forms of cell death within a heterogeneous population of tumor cells [3]. Traditional anti-cancer therapies predominantly impede tumor growth by inducing apoptosis. However, tumor cells can gain resistance to apoptosis through mechanisms such as disruption of the balance between pro-apoptotic and anti-apoptotic proteins, diminished caspase function, and impaired death receptor signaling [4]. Consequently, investigating other forms of cell death may offer complementary or alternative strategies to apoptosis-based therapeutics, potentially enhancing survival and prognosis in cancer patients [5].

Paraptosis is a form of non-apoptotic programmed cell death (PCD) characterized by extensive cytoplasmic vacuolization originating from the endoplasmic reticulum and mitochondrial swelling without the accompanying morphological alterations of apoptosis, autophagy, and necrosis. It typically does not necessitate caspase activation, remains unaffected by Bcl-2 family proteins, and demonstrates insensitivity to apoptosis inhibitors [6, 7]. The induction of paraptosis relies on the synthesis of crucial proteins, endoplasmic reticulum stress, accumulation of misfolded proteins, impaired proteasome function, endoplasmic reticulum Ca^2+^ release and mitochondrial Ca^2+^ overload, and the generation of reactive oxygen species (ROS) [8, 9]. Recently, paraptosis has been proposed as an emerging therapeutic strategy to overcome apoptosis-based drug resistance and effectively hinder the growth of drug-resistant tumors [10–12]. Therefore, we posit that paraptosis may also contribute to cetuximab resistance in CRC.

Focal adhesion kinase (FAK) is a cytoplasmic non-receptor tyrosine kinase with a molecular weight of 125 kDa [13], exhibits aberrant expression in various malignant tumors, including CRC, primary human sarcomas, prostate cancer, breast cancer, ovarian cancer, thyroid cancer, and hepatocellular cancer [13–15]. The overexpression or heightened activity of FAK is closely linked to tumor biological characteristics, including tumorigenesis, proliferation, invasion, metastasis, and resistance to apoptosis [16]. FAK influences the sensitivity of anti-tumor therapy by modulating cell adhesion and suppressing apoptosis through diverse signaling pathways. Through a non-kinase-dependent signaling pathway, FAK interacts with P53 in the nucleus, thereby promoting P53 ubiquitination inactivation and inhibiting tumor cell apoptosis [17]. Additionally, FAK exerts its anti-apoptotic effects through kinase-dependent pathways like the PI3K/AKT signaling [18, 19]. Consequently, FAK emerges as a pivotal factor in mediating resistance to anti-tumor therapy and represents a promising therapeutic target.

Existing approaches to address cetuximab resistance primarily involve investigating novel EGFR-targeted inhibitors, combining multitargeted inhibitors, exploring metabolic regulators, employing immune therapy, developing new cytotoxic drugs [20], and EGFR antibody rechallenge. These approaches predominantly center on optimizing drug utilization, with fewer studies focusing on drug delivery strategies. Exosomes, known for their small size, biocompatibility, and low immunogenicity, provide robust drug protection and exhibit superior tumor-homing ability, allowing for rapid and efficient drug delivery across multiple barriers into cells or tissues [21–24]. Nonetheless, natural exosomes have inadequate targeting properties, rendering them susceptible to rapid clearance and uptake by non-target cells upon entering the body, and interactions with the cell membrane contribute to a shortened half-life of exosomal drugs [25, 26]. What is more essential in clinical practice is an actively targeted drug delivery system such as engineered exosomes capable of achieving precise drug delivery to targeted sites, thereby minimizing the impact on non-target tissues and organs [27]. Targeted peptides or antibody fragments are chemically bonded or genetically engineered onto exosome surfaces to impart cell and tissue specificity. Simultaneously, delivery molecules are loaded into exosomes through intracellular co-expression or electroporation, giving their ability to present specific molecules [28]. Engineered exosomes can precisely load various biologically significant "cargoes," facilitating rapid advancements in biological research and precision medicine. This approach offers novel insights into reversing drug resistance in tumors. The targeted treatment of CRC with the delivery of FAK inhibitors by engineered exosomes may improve tumor sensitivity to EGFR antibodies, reverse drug resistance, and improve efficacy.

In this investigation, we examined the potential function of paraptosis in colon cancer resistant to cetuximab and further explored its intrinsic regulatory mechanisms. Utilizing engineered exosomes, we attempted to deliver target-driving gene siRNAs, observing their impacts on the tumorigenesis and metastasis ability of cetuximab-resistant colon cancer through in vitro and in vivo assays. Our results uncover a novel regulatory mechanism involving paraptosis in cetuximab-resistant colon cancer.

## Materials and methods

### Cell culture

Human-derived 293 T cells and CRC cell lines LIM1215 were procured from Junli Biotech (Shanghai, China) and cultured at 37ºC in an incubator(Thermo Forma, USA) with 5% CO_2_ using DMEM media containing 10% FBS (HyClone, USA). Cetuximab resistance in LIM1215 human colon cancer cell line derivative LIM1215-R was generated by serial passaging with increasing concentrations of cetuximab. Logarithmically growing LIM1215 cells were intermittently exposed to cetuximab for 72 h, followed by recovery in a drug-free medium for 24 h until cells resumed exponential growth, facilitating the selection of drug-resistant derivatives. The concentration range of cetuximab used was 0.001–100 μg/mL.

### Cell counting Kit-8(CCK-8) assay

The CCK-8 (Beyotime, China) was used to measure cell proliferation. 96-well plates were seeded with cells and then added with 10 μL of CCK-8 solution per well and incubated for an hour at 37 °C with 5% CO_2_ humidity. The microplate reader was used to measure the absorbance at 450 nm wavelength to calculate cell viability and the half-maximal inhibitory concentration (IC_50_).

### Colony formation assay

Each group of cells was seeded in Petri dishes at a density of 100 cells/dish and cultured at 37 °C with 5% CO_2_ humidity. Every three days, the medium was replaced, and culturing was terminated upon visible clone formation. Fixed the cells with 4% paraformaldehyde and added Giemsa staining solution (Invitrogen, USA) for 10 ~ 30 min. Clones containing more than 10 cells were observed and counted.

### Real-time quantitative PCR (RT-qPCR)

Trizol and RNA extraction kit (Thermo Fisher, USA) were used to extract total RNA. The nucleic acid concentration and purity were determined using a nucleic acid concentration detector (Themo, USA). Using a PCR instrument (Applied Biosystems, USA) and ReverTra Ace qPCR RT Master Mix (TOYOBO, Japan), reverse transcription reactions were carried out. Quantitative PCR (qPCR) reactions were conducted using SYBR Premix (Thermo Fisher, USA) and a fluorescence quantitative PCR instrument (Applied Biosystems, USA) with the following conditions: 95ºC 5 min → 95ºC 10 s, 60ºC 20 s, 40 cycles → 95ºC 10 s, 60ºC 10 s → 40ºC 30 s. Human glyceraldehyde-3-phosphate dehydrogenase (GAPDH) was selected as the internal reference, and the 2^−ΔΔCt^ method was conducted for data analysis. The primers used in this study are shown in Supplementary Table 1.

### Western blot analysis

Using RIPA lysis buffer (Beyotime, China), the total protein was extracted from each cell group. The BCA method was used to determine the protein concentration. Subsequently, proteins were resolved on SDS‐PAGE and transferred to a PVDF membrane (Millipore, Germany). The blotted membranes were blocked with 5% skim milk for 2 h at room temperature and were incubated with primary antibodies (AFT3 1:2000, IRE1α 1:1000, pIRE1 1:1000, CHOP 1:1500, FAK 1:1000, Phospho-FAK 1:1000, CAV1 1:1000, PAK3 1:2000, PIK3R3 1:2000) at 4 °C overnight. Following three TBST washes, the membrane was incubated for 1 h at room temperature with a secondary antibody diluent. Protein quantification was performed after ECL luminescence imaging. The antibodies used in this study are shown in Supplementary Table 2.

### Immunofluorescence (IF) staining

After being fixed with 4% paraformaldehyde, cells were permeabilized in 0.1% Triton-X-100 (Bio-Rad, USA) and blocked for an hour at room temperature using PBS containing 3% BSA. The primary antibody was then incubated with the cells overnight at 4 °C in a humidified environment. After adding fluorescent secondary antibodies, the cells were incubated for 1 h in the dark. To stain the cells, DAPI (Sigma, USA) was utilized. After sealing the slides, using a laser confocal microscope (Zeiss, Germany) to observe. The antibodies used in this study are shown in Supplementary Table 2.

### RNA-sequencing (RNA-seq)

The TRIzol reagent was used to extract total RNA under the instructions. A NanoDrop 2000 spectrophotometer (Thermo Scientific, USA) was used to determine RNA purity and quantification. An Agilent 2100 Bioanalyzer (Agilent Technologies, USA) was used to evaluate RNA integrity. The instructions for the VAHTS Universal V5 RNA-seq Library Prep kit were followed to construct transcriptome libraries. Shanghai Ouyi Biotechnology Co (Shanghai, China) conducted transcriptome sequencing and analysis. lllumina Novaseq 6000 sequencing platform was used to sequence the library and 150 bp double-ended reads were generated. For every sample, approximately 50 M raw reads were acquired. The fastp [29] software was used to process the fastq format raw reads, and the clean reads were obtained for further data processing after deleting the low-quality reads. The reference genome comparison was performed using the HISAT2 [30] software, gene expression level (FPKM) [31] was computed, and HTSeq-count [32] was used to acquire the counts of reads for each gene. R (v3.2.0) was used for PCA analysis of genes (counts) and mapping to evaluate sample biological replicates. DESeq2 [33] software was used to analyze differentially expressed genes (DEGs). Genes that satisfied q value < 0.05 and foldchange > 2 or foldchange < 0.5 threshold were classified as DEGs. To show the gene expression patterns across groups and samples, R (v 3.2.0) was used to perform the hierarchical clustering analysis of DEGs. To illustrate the shift of up- or down-regulated gene expression, radar plots were created for the top 30 genes using the R package. Following this, enrichment analyses were conducted on the differentially expressed genes using the hypergeometric distribution algorithm. The analyses included GO [34], KEGG [35] Pathway, Reactome, and WikiPathways, aiming to identify significantly enriched functional entries, and bar charts, chord charts, and enrichment analysis circle plots were drawn by R (v 3.2.0).

### Transfection of FAK siRNA

Cells were transfected using the lipofectamine RNAi MAX reagent (Thermo Fisher, USA). Briefly, siRNAs (NC siRNA/FAK siRNA) were separately incubated with Lipofectamine in Opti-MEM medium (Gibco, USA) for 20 min. After that, the cells were incubated for six hours in the culture plates with the siRNAs matrix added. Transfected cells were then utilized for cellular morphology observation, colony formation assay, CCK-8 assay, RT-qPCR, as well as Western Blot to assess both gene and protein expression levels. NC siRNA/FAK siRNA sequence:

- NC siRNA: 5’-GAUAAGCCGAUACCUACUA-3’
- FAK siRNA: 5’-GGACGAAACAGAUGAUUAU-3’

### Construction and morphological observation of engineered exosomes

#### Exosomes collection and extraction

A pLVX-Her2 binding affibody-LAMP2 vector sequence was constructed and transfected into 293 T cells cultured in T75 flasks. To remove bigger vesicles, 1L of supernatant from grown 293 T cells was centrifuged for 30 min at 2000 × g, followed by 10,000 × g for 45 min (Beckman, USA). Retrieve the supernatant, filter through a 0.45 μm membrane (Millipore, Germany), collect the filtrate, and centrifuge for 70 min at 100,000 × g (Hitachi, Japan). Remove the supernatant, resuspend in 10 mL pre-chilled 1 × PBS, and ultra-centrifuge at 100,000 × g for 70 min. Resuspend in 70 μL pre-chilled 1 × PBS after removing the supernatant again. All centrifugation steps were carried out at 4℃. Her2 binding affibody sequence: (VDNKFNKEMRNAYWEIALLPN LN NQ QK R A FI R SLYDDP SQ SA N L L A E A KKLNDAQAPK).

#### Characterization Analysis of Exosomes

Diluted exosomes were subjected to particle size determination using a nanoparticle tracking analyzer (ZetaVIEW, Particle Metrix, Germany).

Exosome morphology was observed using a transmission electron microscope (HT-7700, Hitachi, Japan): 10μL exosome sample was dropped onto a copper grid and given a minute to settle. The filter paper was used to remove the extra liquid. Then,10μL of uranyl acetate was dropped onto the copper grid and settled for 1 min, followed by removing the excess liquid. The grid underwent several minutes of air drying at room temperature before being detected using transmission electron microscopy at 100 kV.

Cellular Uptake of Exosomes: PKH67, a green fluorescent dye, was used to label engineered exosomes. LIM1215 and LIM1215-R cells were separately co-incubated with the stained exosomes. Hoechst staining solution was employed to label cell nuclei. Observation and imaging were performed under a fluorescence microscope.

#### Exosome Loading with FAK siRNA

FAK siRNA was loaded into isolated exosomes using an electroporation system (Neon NxT Electroporation System, Thermo Fisher, 800 V, 10 ms, 3 pulses). The loading dosage was 500 nmol of FAK siRNA per 10 μg of exosomes.

### Animal experiments

#### Tumor Xenograft

Eight-week-old female BALB/c nude mice were available from Cavens Experimental Animal Co., Ltd. (Changzhou, China) and divided into four groups randomly, with five mice in each. Take 10^7^ tumor cells from each group and subcutaneously implant them into the corresponding groups of mice in the axillary region to establish a xenograft model. From the fifth day onward, a total of seven times, engineered exosomes (CT-Exo-siNC/CT-Exo-siFAK1 Injection, 100 μg/mice, i.v.) were given every five days. For a total of four injections every ten days, cetuximab (1 mg/kg, i.p.) was given. Tumor size curves were recorded every 5 days until day 40. Finally, the mice were sacrificed to measure the tumor volume and weight. Tumor sections were then used for RT-qPCR and IF staining.

#### Metastasis model

10^6^ tumor cells from each group were intrasplenically injected into 20 eight-week-old female BALB/c nude mice respectively, with 5 mice in each group, to establish a liver metastasis model. The mice were treated with engineered exosomes and cetuximab using the aforementioned methods. The mice were sacrificed after 40 days, and liver tissues were taken. Then, counted the visible liver metastatic nodules of every mouse, and the tumor section underwent H&E and IF staining. All animal experiments were conducted at the Animal Experimental Center of Soochow University and approved by the Ethics Committee for Animal Experiments of the Third Affiliated Hospital of Soochow University.

### Tissue staining

After fixing with 4% paraformaldehyde, the tumor tissues were dehydrated, embedded, and sectioned into 5-μm slices. After deparaffinization and hydration, high-temperature antigen retrieval was performed. For 10 min, each slide was treated with 1 drop or 50 μL of hydrogen peroxide blocking solution at room temperature. At 4 °C, primary antibodies were added to slices and incubated overnight. Fluorescent secondary antibodies were then added at room temperature for 10 min. After DAPI staining for 10 min, the results were observed under a fluorescence microscope (OLYMPUS, Japan), and images were captured using a laser confocal microscope (Zeiss, Germany). H&E stain was also applied to tumor slices.

### Statistical analysis

Statistical analysis and graphical representation were performed using GraphPad Prism 8.0. The data are expressed as "Mean ± SD." Student's t-test was employed to assess differences between the two groups, while two-way ANOVA was utilized for statistical comparisons involving more than two groups. *P < 0.05, **P < 0.01, and ***P < 0.001.

## Results

### Paraptosis was suppressed in cetuximab-resistant *colon cancer* cells

**Fig. 1:**
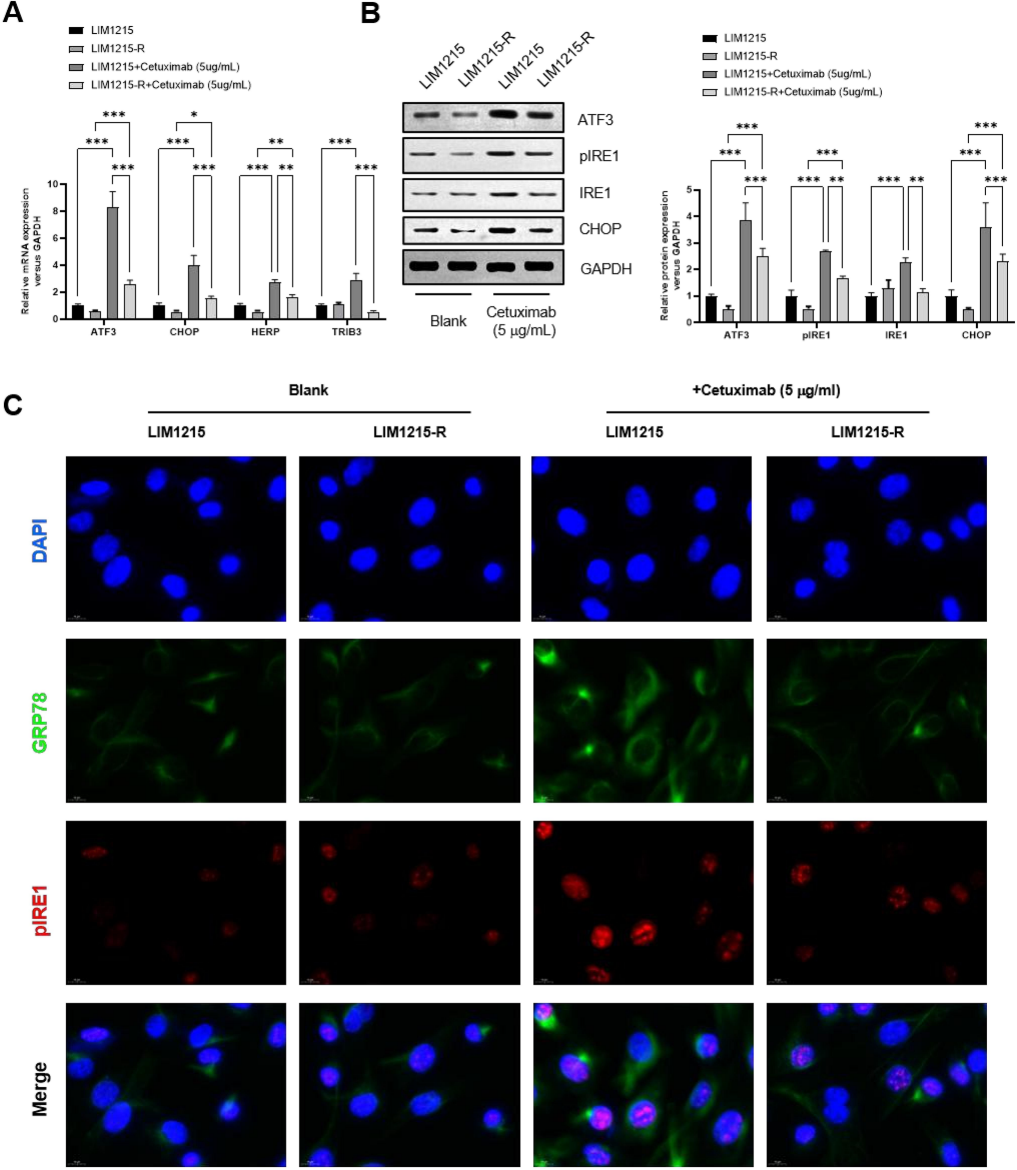
Paraptosis was suppressed in cetuximab-resistant colon cancer cells. **A** The mRNA expression levels of paraptosis-related genes (ATF3, CHOP, HERP, and TRIB3) were analyzed by real-time PCR. **B** The protein expression levels of paraptosis-related genes (ATF3, pIRE1, IRE1, and CHOP) were analyzed by Western Blot. **C** The protein expression levels of paraptosis-related genes (GRP78 and pIRE1) were verified by IF staining. *P < 0.05, **P < 0.01, ***P < 0.001, versus indicated group, *n* = 3

To validate the involvement of paraptosis in cetuximab resistance in CRC, we induced resistance in the CRC cell line LIM1215 by gradually increasing the concentration of cetuximab until reaching a concentration of 50 μg/mL, thereby establishing the cetuximab-resistant cell line LIM1215-R. Using regular LIM1215 cells as a control, we examined differences in paraptosis-related indicators. The results demonstrated that after treatment with 5 μg/mL cetuximab for 12 h, the expression levels of paraptosis-related genes ATF3, CHOP, HERP, and TRIB3 mRNA (Fig. 1A) and ATF3, pIRE1, IRE1, and CHOP proteins (Fig. 1B) were significantly upregulated in LIM1215 cells. In contrast, the upregulation of paraptosis-related gene mRNA and protein levels in resistant LIM1215-R cells was markedly attenuated compared to LIM1215 cells (Fig. 1A-B). Furthermore, IF revealed that, following cetuximab treatment, the protein expression levels of paraptosis-related genes GRP78 and p-IRE1 were more significantly upregulated in sensitive cells compared to resistant cells, as shown in Fig. 1C. In summary, cetuximab can induce paraptosis in CRC cells, with a weakened induction of paraptosis observed in resistant cells. These data indicate the suppression of paraptosis in cetuximab-resistant colon cells.

### Paraptosis inhibition promoted the cetuximab-resistance in *colon cancer* cells

**Fig. 2:**
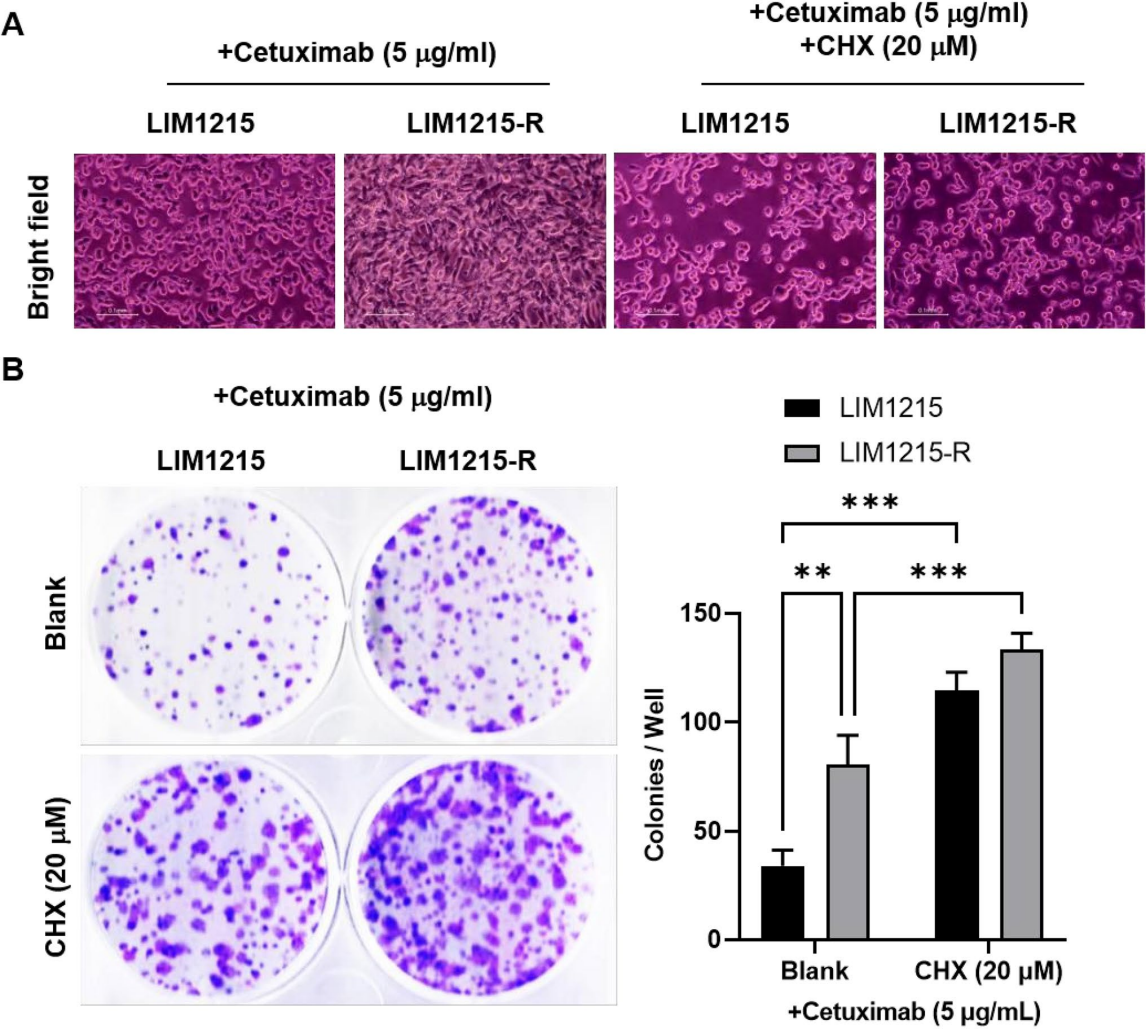
Paraptosis inhibition promoted the cetuximab-resistance in colon cancer cells. **A **Representative cell morphology was shown. **B** The LIM1215 and LIM1215-R cells formatted colonies with or without paraptosis inhibitor (CHX, 20 mM) were shown. **P < 0.01, ***P < 0.001, versus indicated group, *n* = 3

To delve deeper into the role of paraptosis in cetuximab resistance, both cell types were pre-treated with the protein synthesis inhibitor cycloheximide (CHX). Cell proliferation capacity was assessed through morphological observations and colony formation experiments. The results revealed that after 12 h of treatment with 5 μg/mL cetuximab, LIM1215-R cells exhibited denser growth and stronger colony-forming ability with a higher number of formed colonies compared to LIM1215 cells. Additionally, the addition of 20 μM CHX not only enhanced the growth capacity of LIM1215 cells but also further promoted proliferation in LIM1215-R cells, as depicted in Fig. 2A-B. This suggests that the addition of the paraptosis inhibitor CHX can promote resistance to cetuximab in both LIM1215 and LIM1215-R cells. Furthermore, after 12 h of cetuximab and CHX treatment, the expression levels of paraptosis-related gene mRNA and proteins were significantly downregulated in LIM1215 cells, while there was no apparent change in paraptosis indicators in LIM1215-R cells (Supplementary Fig. 1A-C). This indicates that the inhibition of paraptosis may be one of the mechanisms mediating cetuximab resistance.

### Focal adhesion kinase (FAK) signaling pathway was activated in cetuximab-resistant *colon cancer* cells.

**Fig. 3:**
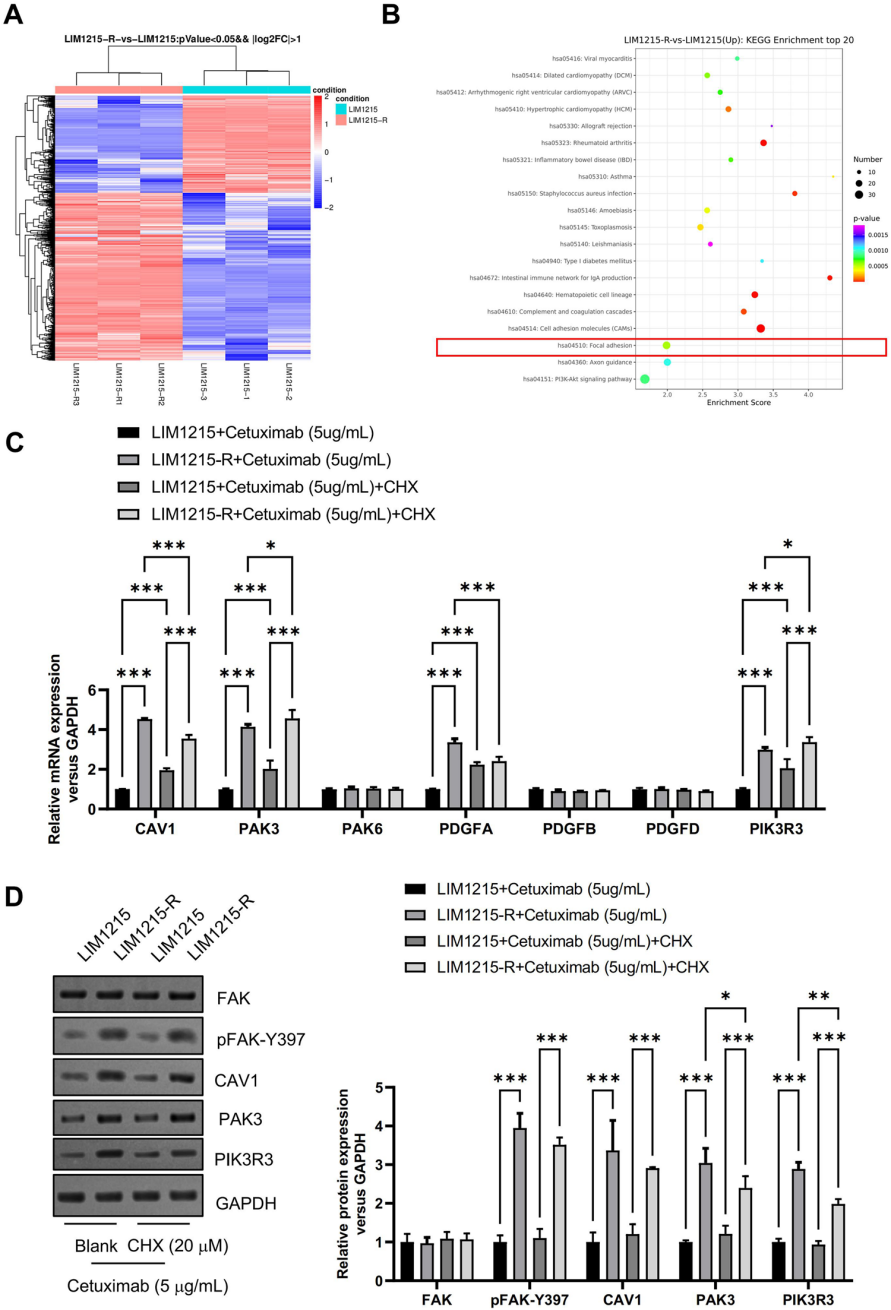
Focal adhesion kinase (FAK) signaling pathway was activated in cetuximab-resistant colon cancer cells. (**A**) Transcriptomics sequencing and (**B**) Kyoto Encyclopedia of Genes and Genomes (KEGG) analysis were performed to evaluate the dysregulated genes and signaling pathways. (**C**) The mRNA expression levels of FAK signaling dysregulated genes (CAV1, PAK3, PAK6, PDGFA, PDGFB, PDGFD, and PIK3R3) were analyzed by real-time PCR. (**D**) The protein expression levels of FAK signaling dysregulated genes (FAK, pFAK-Y397, CAV1, PAK3, and PIK3R3) were analyzed by Western Blot. *P < 0.05, **P < 0.01, ***P < 0.001, versus indicated group, *n* = 3

A comparative analysis of mRNA expression and signaling pathways differences between LIM1215-R and LIM1215 cells was conducted through RNA-seq, as illustrated in Fig. 3A-B. Kyoto Encyclopedia of Genes and Genomes (KEGG) pathway enrichment analysis indicated that differentially expressed genes were primarily involved in signaling pathways such as cell adhesion molecules, PI3K-Akt signaling pathway, focal adhesion, and intestinal immune network for IgA production (Fig. 3B). As previously mentioned, FAK regulates cell adhesion and inhibits apoptosis through various signaling pathways, influencing the sensitivity of anti-tumor therapy. Therefore, FAK may be a key factor in mediating cetuximab resistance in CRC.

To validate this hypothesis, differences in the expression of FAK signaling dysregulated genes in LIM1215 and LIM1215-R cells were examined through RT-qPCR and Western Blot. The results demonstrated that in LIM1215 cells, after 12 h of treatment with cetuximab (5 μg/mL) and the paraptosis inhibitor CHX (20 μM), the mRNA expression levels of FAK signaling dysregulated genes (CAV1, PAK3, PDGFA, and PIK3R3) were upregulated (Fig. 3C). In contrast, regardless of CHX addition, the protein expression levels of FAK signaling dysregulated genes (pFAK-Y397, CAV1, PAK3, and PIK3R3) were naturally higher in cetuximab-resistant LIM1215-R cells compared to LIM1215 cells (Fig. 3D). Phosphorylation of FAK at Y397 is a crucial step in activating its catalytic activity and signaling from focal adhesions [36]. As shown in Fig. 3D, in both CHX-treated and untreated conditions, the phosphorylation level of pFAK-Y397 in LIM1215-R cells, following cetuximab treatment, was significantly elevated compared to LIM1215 cells. These data suggest a significantly heightened activation of the FAK signaling pathway in cetuximab-resistant colon cancer cells.

### FAK knockdown inhibited cetuximab resistance and activated the paraptosis in *colon cancer* cells.

**Fig. 4:**
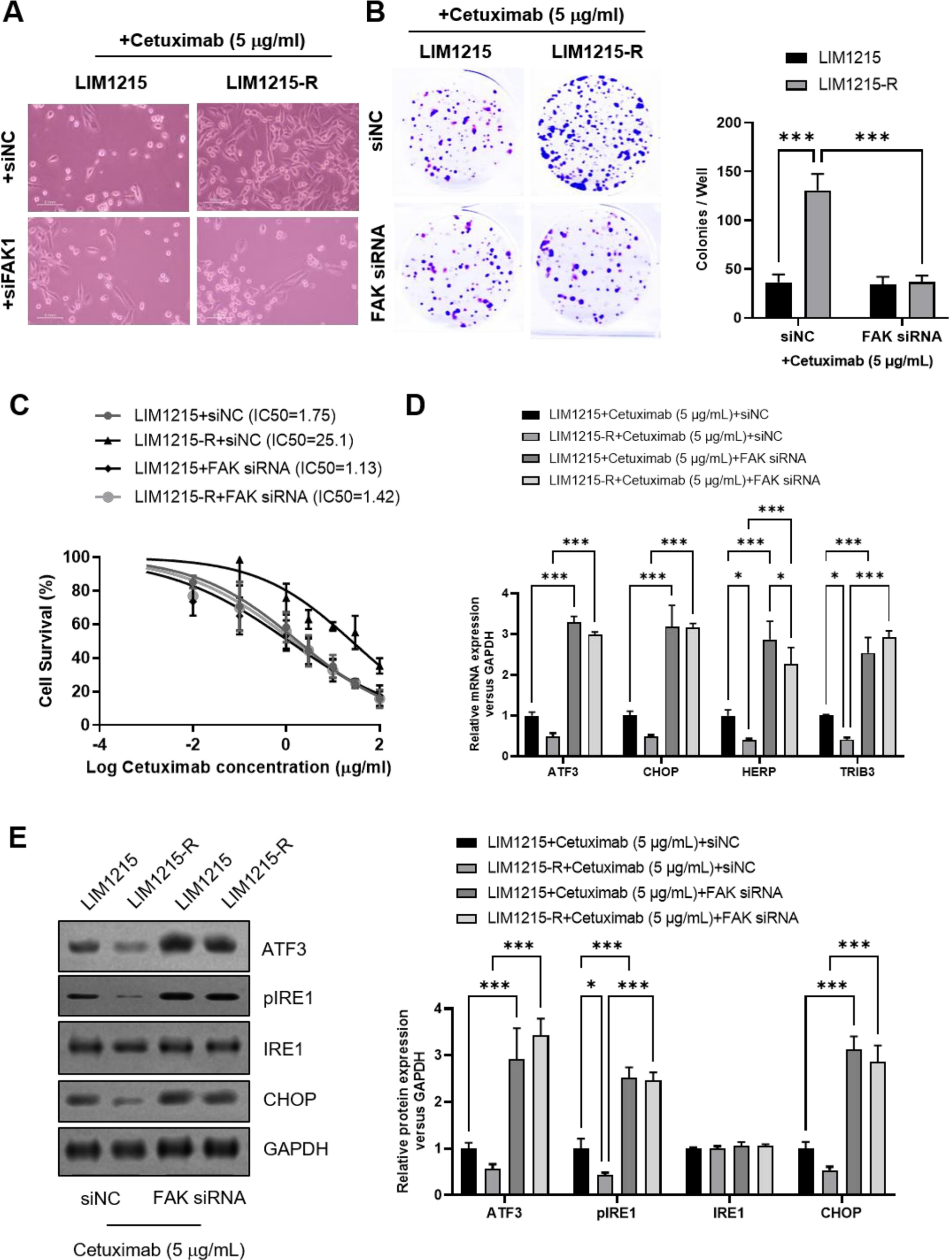
FAK knockdown inhibited cetuximab resistance and activated the paraptosis in colon cancer cells. **A** Representative cell morphology was shown. **B** The LIM1215 and LIM1215-R cells formatted colonies with or without FAK knockdown were shown. **C** The IC_50_ values of cetuximab in LIM1215 and LIM1215-R cells with or without FAK knockdown were analyzed by CCK-8 assay. **D** The mRNA expression levels of paraptosis-related genes (ATF3, CHOP, HERP, and TRIB3) were analyzed by real-time PCR. **E** The protein expression levels of paraptosis-related genes (ATF3, pIRE1, IRE1, and CHOP) were analyzed by Western Blot. *P < 0.05, **P < 0.01, ***P < 0.001, versus indicated group, *n* = 3

To further explore the correlation between FAK activation and paraptosis in cetuximab resistance, we transfected FAK siRNA and assessed differences in cell phenotype and paraptosis-related indicators. The results revealed that under cetuximab treatment (5 μg/mL), LIM1215-R cells in the si-NC group exhibited higher viability, a more extended morphology, and increased clonogenicity, forming larger and denser colonies. Transfection of FAK siRNA significantly inhibited the activity and proliferation of LIM1215-R cells (Fig. 4A-B). Similarly, CCK-8 assays demonstrated that compared to the si-NC group, transfection of FAK siRNA significantly reduced the IC50 of LIM1215-R cells under different concentrations of cetuximab treatment (Fig. 4C), indicating the disappearance of the drug-resistant phenotype after FAK downregulation. This underscores the critical role of the FAK signaling pathway in cetuximab resistance in CRC. Furthermore, we investigated the impact of FAK on paraptosis by examining the expression of paraptosis-related indicators. RT-qPCR results indicated that compared to the si-NC group, transfection of FAK-siRNA significantly increased the mRNA levels of paraptosis-related indicators (ATF3, CHOP, HERP, TRIB3) in both LIM1215 and LIM1215-R cells (Fig. 4D). The results of Western Blot further confirmed that in both cell lines, FAK siRNA transfection markedly increased the protein expression of genes associated with paraptosis (ATF3, pIRE1, and CHOP) (Fig. 4E). These findings suggest that inhibiting FAK can activate paraptosis, simultaneously affecting the phenotype of cetuximab-resistant cells.

### Isolation and characterization of engineered *colon cancer* cells targeted and FAK siRNA loaded exosomes.

**Fig. 5:**
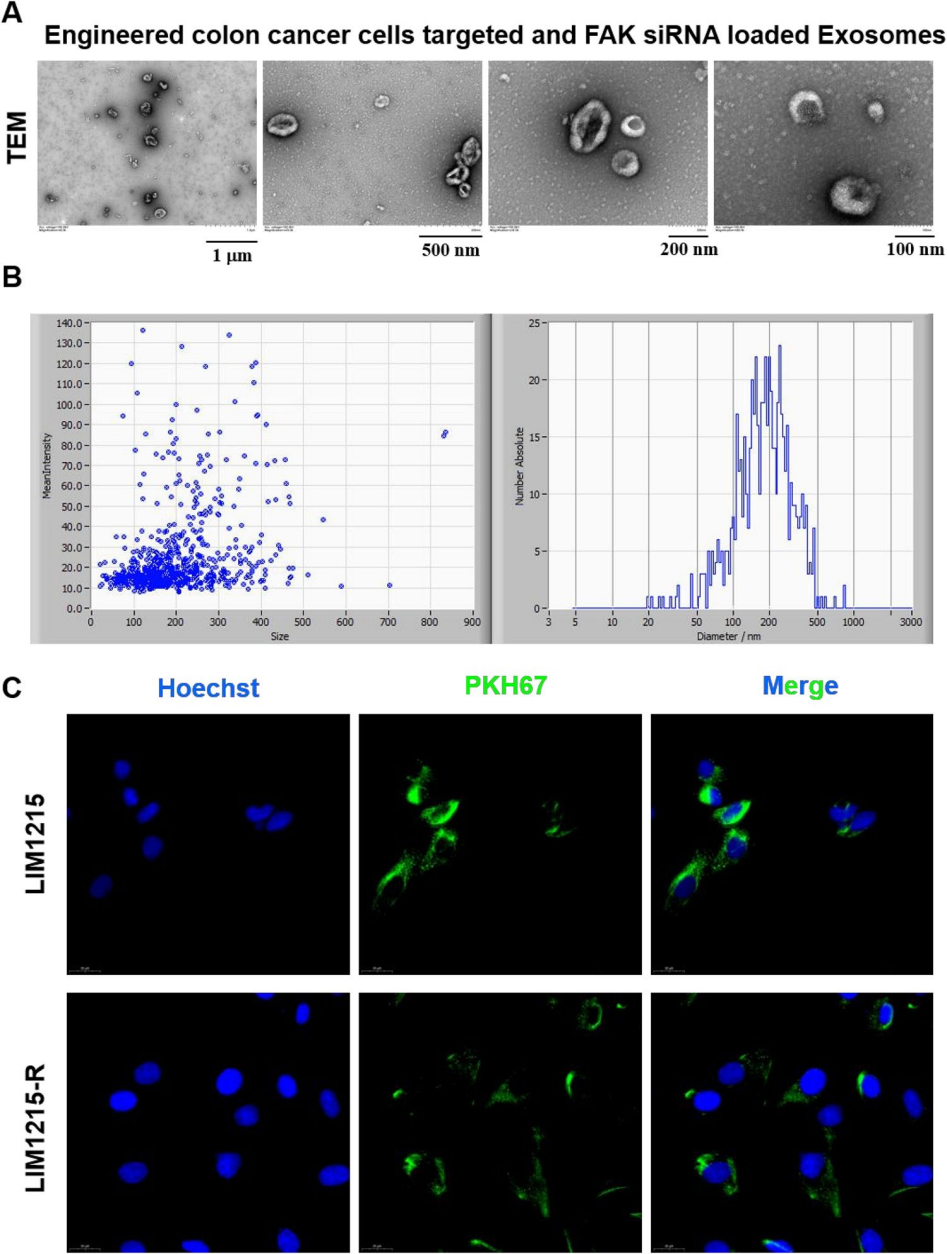
Isolation and characterization of engineered colon cancer cells targeted and FAK siRNA loaded exosomes. **A** Transmission electron micrograph of the engineered colon cancer cells targeted and FAK siRNA loaded exosomes (CT-Exo-siFAK1). **B** The size distribution of the CT-Exo-siFAK1 was measured by NTA. **C** The Uptake of the PKH67 labeled CT-Exo-siFAK1 by LIM1215 and LIM1215-R cells was shown by a Fluorescence microscope

By constructing a pLVX-Her2 binding affibody-LAMP2 vector sequence targeting HER-2 expressing cells, we subsequently transfected this vector sequence into 293 T cells. Exosomes were then isolated from the culture supernatant using ultracentrifugation. FAK siRNA was loaded into these exosomes using electroporation, creating engineered colon cancer cells targeted and FAK siRNA-loaded exosomes (CT-Exo-siFAK1) (Fig. 9). Morphological and size assessments of the exosomes were conducted using Transmission Electron Micrograph (TEM) and Nanoparticle Tracking Analysis (NTA). The results demonstrated that CT-Exo-siFAK1 exhibited a typical dish-shaped double-layered membrane structure, with uniform size and an approximate diameter of 150 nm (Fig. 5A-B). Subsequently, CT-Exo-siFAK1 was labeled with the fluorescent dye PKH67, and PKH67-CT-Exo-siFAK1 was separately cultured with LIM1215 and LIM1215-R cells to verify the uptake capacity of both cell types. Fluorescence microscope results indicated that CT-Exo-siFAK1 could effectively enter both LIM1215 and LIM1215-R cells (Fig. 5C).

### CT-Exo-siFAK1 inhibited cetuximab resistance and activated the paraptosis in *colon cancer* cells in vitro.

**Fig. 6:**
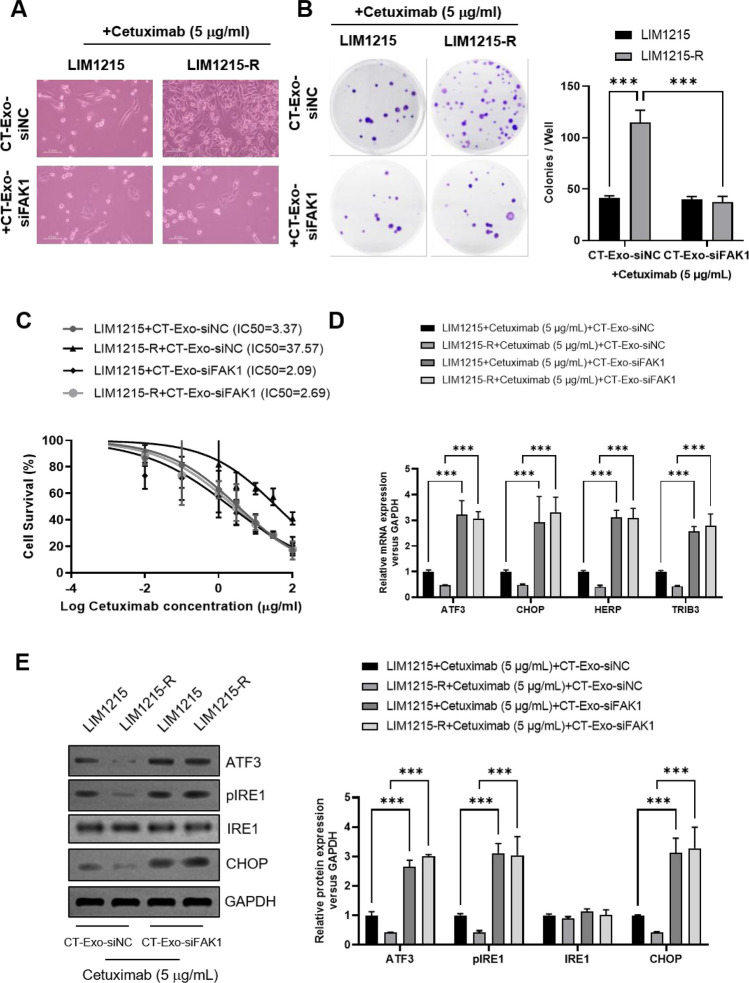
CT-Exo-siFAK1 inhibited cetuximab resistance and activated the paraptosis in colon cancer cells in vitro. **A** Representative cell morphology was shown. **B** The LIM1215 and LIM1215-R cells formatted colonies with or without CT-Exo-siFAK1 treatment were shown. **C** The IC_50_ values of cetuximab in LIM1215 and LIM1215-R cells with or without CT-Exo-siFAK1 treatment were analyzed by CCK-8 assay. **D** The mRNA expression levels of paraptosis-related genes (ATF3, CHOP, HERP, and TRIB3) were analyzed by real-time PCR. **E** The protein expression levels of paraptosis-related genes (ATF3, pIRE1, IRE1, and CHOP) were analyzed by Western Blot. *P < 0.05, **P < 0.01, ***P < 0.001, versus indicated group, *n* = 3

To explore the impact of CT-Exo-siFAK1 on proliferation in both cell lines, we conducted cellular morphology observation, colony formation assays, and CCK-8 analysis. After 12 h of cetuximab treatment, compared to the control group, the viability of LIM1215-R cells co-cultured with CT-Exo-siFAK1 significantly decreased (Fig. 6A), and the number of formed colonies markedly reduced (Fig. 6B). Simultaneously, the IC_50_ was significantly lower than the control group (Fig. 6C). These results indicate that CT-Exo-siFAK1 can effectively deliver FAK siRNA to recipient cells and inhibit the proliferation of cetuximab-resistant LIM1215-R cells. To further confirm the biological effects induced by CT-Exo-siFAK1, we evaluated the mRNA and protein levels of paraptosis-related genes. As expected, compared to cells treated with CT-Exo-siNC, LIM1215-R cells treated with CT-Exo-siFAK1 showed an upregulation in the mRNA expression levels of ATF3, CHOP, HERP, and TRIB3 (Fig. 6D). Similarly, the protein expression levels of paraptosis-related genes (ATF3, pIRE1, and CHOP) increased (Fig. 6E). These results indicate that in vitro, CT-Exo-siFAK1 can effectively activate paraptosis in colon cells and inhibit cetuximab resistance.

### CT-Exo-siFAK1 activated paraptosis and inhibited the tumorigenesis ability of cetuximab-resistant *colon cancer* cells in vivo.

**Fig. 7:**
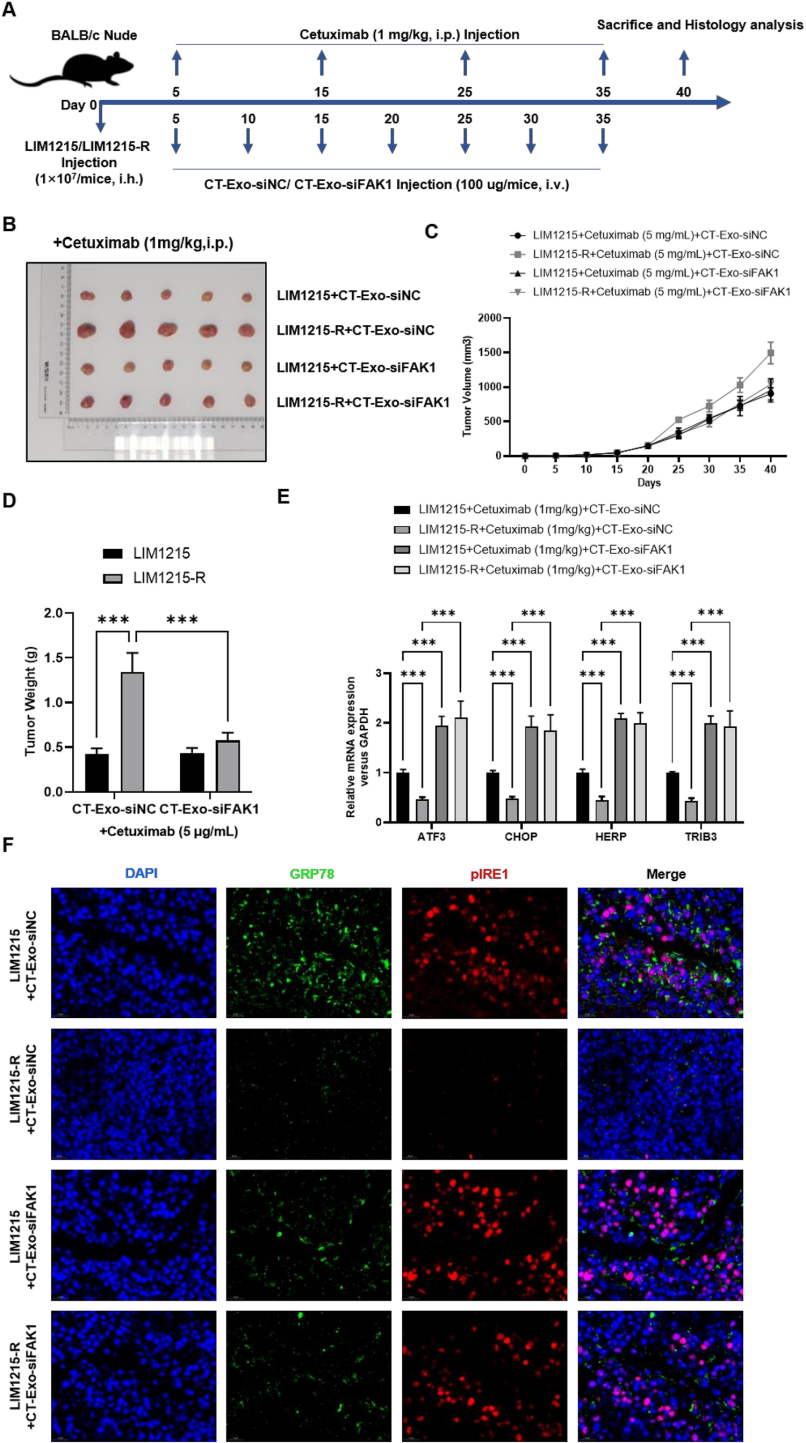
CT-Exo-siFAK1 activated paraptosis and inhibited the tumorigenesis ability of cetuximab-resistant colon cancer cells in vivo. **A** Experimental timeline showing the timing of LIM1215/LIM1215 cells, Cetuximab, and CT-Exo-siNC/ CT-Exo-siFAK1 injection, as well as the timing for mice sacrifice and histology analysis. **B** Representative image of the LIM1215/LIM1215 cells generated tumors and the **C** tumor volume and **D** tumor weight was shown. (E) The mRNA expression levels of paraptosis-related genes (ATF3, CHOP, HERP, and TRIB3) were analyzed by real-time PCR. **E** The protein expression levels of paraptosis-related genes (GRP78 and pIRE1) were analyzed by IF staining. ***P < 0.001, versus indicated group, *n* = 5

To investigate the effects of CT-Exo-siFAK1 on cetuximab-resistant colon cancer in vivo, we induced subcutaneous tumor models in BALB/c female nude mice by injecting 1 × 10^7^ doses of LIM1215 or LIM1215-R cells. Five days later, mice were intravenously injected with CT-Exo-siFAK1 or CT-Exo-siNC (100 μg/each), once every 5 days for 40 days. Additionally, cetuximab was intraperitoneally injected at 1 mg/kg every 10 days (Fig. 7A). Tumor size curves were recorded every 5 days until day 40. Finally, tumors were excised from the mice to measure volume and weight, and histological analyses were performed. As shown in Fig. 7B-D, compared to the CT-Exo-siNC group, tumor growth in LIM1215-R mice injected with CT-Exo-siFAK1 was significantly inhibited, with reduced tumor volume and number. These results suggest that CT-Exo-siFAK1 can increase the sensitivity of colon cancer cells to cetuximab in vivo. To further evaluate the induced paraptosis in tumors by CT-Exo-siFAK1, we assessed the regulatory activity of paraptosis-related genes through RT-qPCR and IF staining. As shown in Fig. 7E, CT-Exo-siFAK1 upregulated the mRNA expression of ATF3, CHOP, HERP, and TRIB3 in tumor tissues. IF also demonstrated that CT-Exo-siFAK1-treated mice exhibited increased protein expression of GRP78 and p-IRE1 (Fig. 7F). These in vivo experimental results are consistent with the in vitro findings, providing strong evidence that CT-Exo-siFAK1 activates paraptosis in cetuximab-resistant colon cancer cells in vivo, thereby inhibiting their tumorigenicity.

### CT-Exo-siFAK1 activated paraptosis and inhibited the metastasis ability of cetuximab-resistant *colon cancer* cells in vivo.

**Fig. 8:**
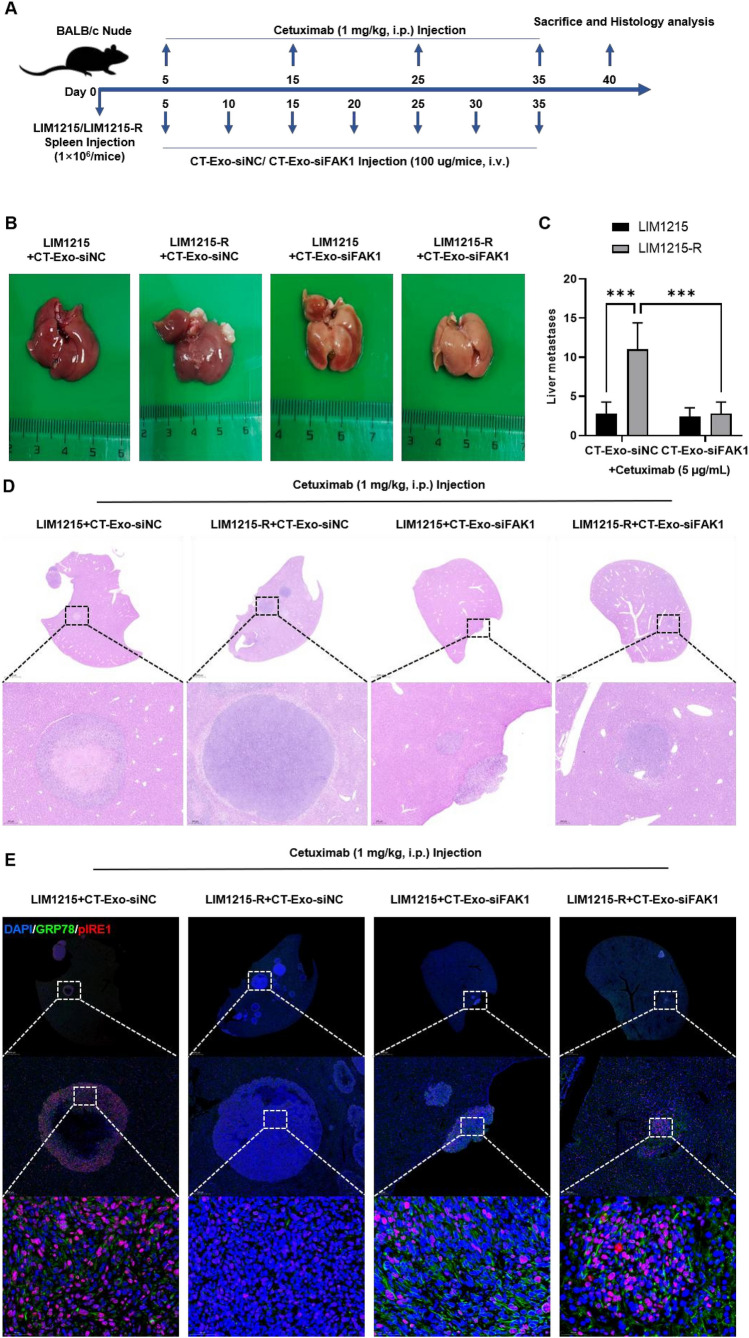
CT-Exo-siFAK1 activated paraptosis and inhibited the metastasis ability of cetuximab-resistant colon cancer cells in vivo. **A** Experimental timeline showing the timing of LIM1215/LIM1215 cells, Cetuximab, and CT-Exo-siNC/ CT-Exo-siFAK1 injection, as well as the timing for mice sacrifice and histology analysis. **B** The representative images of the LIM1215/LIM1215 cells generated liver metastases and the **C** calculated number was shown. **D** H&E staining was performed to show the metastases focus of colon cancer cells. **E** The protein expression levels of paraptosis-related genes (GRP78 and pIRE1) were analyzed by IF staining in the metastases focus of colon cancer cells. ***P < 0.001, versus indicated group, *n* = 5

To explore the role of CT-Exo-siFAK1 in tumor metastasis in vivo, we initially established a murine liver metastasis model by splenic injection of LIM1215/LIM1215-R cells. Mice were treated with CT-Exo-siFAK1/CT-Exo-siNC (100 μg/each, intravenous injection) and cetuximab (1 mg/kg, intraperitoneal injection). Tumor size curves were recorded, and liver metastases were excised at last for weighing and histological analysis (Fig. 8A). The results demonstrated that compared to the control group, CT-Exo-siFAK1 treatment significantly reduced the number and size of liver metastatic lesions produced by LIM1215-R cells (Fig. 8B-C), with a noticeable decrease of tumors in H&E-stained liver sections (Fig. 8D). Further investigation revealed that in the metastatic foci within the LIM1215-R/CT-Exo-siFAK1 group, the paraptosis-related genes GRP78 and pIRE1 showed a significant elevation in protein expression levels in comparison to the control group (Fig. 8E). According to these results, CT-Exo-siFAK1 not only activates paraptosis in cetuximab-resistant colon cancer cells but also inhibits their metastatic potential in vivo.

## Discussion

In the treatment of advanced CRC, cetuximab plays a crucial role. Nevertheless, the development of resistance poses a significant obstacle to effective therapy, underscoring the need to investigate promising approaches to address this challenge. The primary goal of many cancer therapies is to eradicate malignant cells by triggering apoptosis and associated cell death pathways. Nevertheless, cancer cells employ various anti-apoptotic mechanisms, allowing them to evade apoptosis and leading to drug resistance, tumor survival, and recurrence [37]. Inducing non-apoptotic cell death is gaining recognition as a potential approach in anti-tumor treatment. Paraptosis, characterized by cytoplasmic vacuolation, is a recently discovered form of non-apoptotic PCD that plays a crucial role in cancer. For example, the potential of Elaiophylin-induced paraptosis lies in its ability to overcome platinum, taxane, and PARP inhibitor resistant ovarian cancer through the over-activation of the SHP2/SOS1/MAPK pathway [11]. Combining the proteasome inhibitor (PI) bortezomib (Btz) with loperamide could enhance endoplasmic reticulum stress and expansion meditated by Btz, overcoming colon cancer cell resistance to PIs by inducing paraptosis-like cell death [10]. Despite shedding new light on cancer treatment perspectives, it has not been investigated if paraptosis is involved in cetuximab resistance in CRC. Paraptosis can be induced by the human insulin-like growth factor 1 receptor (IGF-1R) [6], and the IGF-1/IGF-1R pathway is essential for the angiogenesis, migration, apoptosis, proliferation, and differentiation of CRC. Overactivation of this pathway, leading to upregulation of the PI3K/AKT pathway, is implicated in primary and acquired resistance of RASwt mCRC to EGFR inhibitors [38, 39]. Therefore, we hypothesize that paraptosis may also be one of the mechanisms underlying resistance to cetuximab in CRC.

This study reveals that, compared to the common sensitive LIM1215 cells, the level of cetuximab-induced paraptosis is downregulated in resistant colon cancer cells. The addition of the protein synthesis inhibitor CHX promotes cetuximab resistance in LIM1215 cells and downregulates the expression of paraptosis-related indicators. However, the paraptosis level in the resistant LIM1215-R cells does not exhibit further downregulation after CHX treatment. This could be attributed to the inherently low paraptosis level in LIM1215-R cells, making it difficult to observe significant differences following CHX inhibition. Interestingly, the clonogenic capacity of LIM1215-R cells increases after CHX treatment. This suggests that the suppression of paraptosis is just one of the multiple mechanisms contributing to cetuximab resistance in CRC. Other factors, such as aberrant activation of EGFR-related signaling pathways in tumor cells, genomic instability such as genetic or epigenetic alterations [40], changes in cell abundance, and cytokine within the tumor microenvironment, may also constitute integral components of the regulatory network associated with cetuximab resistance [41].

Through KEGG analysis, the FAK signaling pathway was identified as a key pathway involved in inhibiting paraptosis. FAK, a cytoplasmic functional protein, acts through kinase-dependent mechanisms. Its activation targets multiple downstream signaling pathways, regulating various cellular functions, including tumorigenesis, proliferation, invasion, metastasis, and anti-apoptosis, making it a critical factor in anti-cancer treatment resistance. For example, downregulation of FAK can enhance the radiosensitivity of colon cancer cells [42]. Combining FAK inhibitors with KRAS G12C inhibitors may reduce resistance to KRAS G12C inhibitors by targeting dysregulated FAK-YAP signaling and fibrogenesis [43]. Co-delivery of paclitaxel and FAK siRNA has been shown to increase cytotoxicity and apoptosis in paclitaxel-resistant tumors [44]. FAK inhibitors have also been identified to enhance the chemosensitivity of 5-FU in gastric cancer by activating p53 transcriptional activity, serving as a valuable prognostic indicator [45]. Therefore, cancer treatment targeting FAK has become a research hotspot and is a potential strategy to overcome cancer treatment resistance. This study reveals the activation of the FAK signaling pathway in colon cancer cells resistant to cetuximab. Knocking out FAK upregulates the expression of paraptosis-related indicators in both LIM1215 and LIM1215-R cells and reverses the phenotype of resistant cells. Notably, in LIM1215 cells, despite the upregulation of paraptosis-related gene expression upon FAK knockout, there is no significant difference in clonal formation and proliferation compared to the control group. This is likely due to the inherently low clonal levels in the sensitive strain after cetuximab treatment. Therefore, following FAK downregulation, there was no apparent distinction in clonal formation and proliferation compared with the control group. In summary, these findings suggest that FAK knockout can activate paraptosis, thereby reversing cetuximab resistance in colon cancer.

**Fig. 9:**
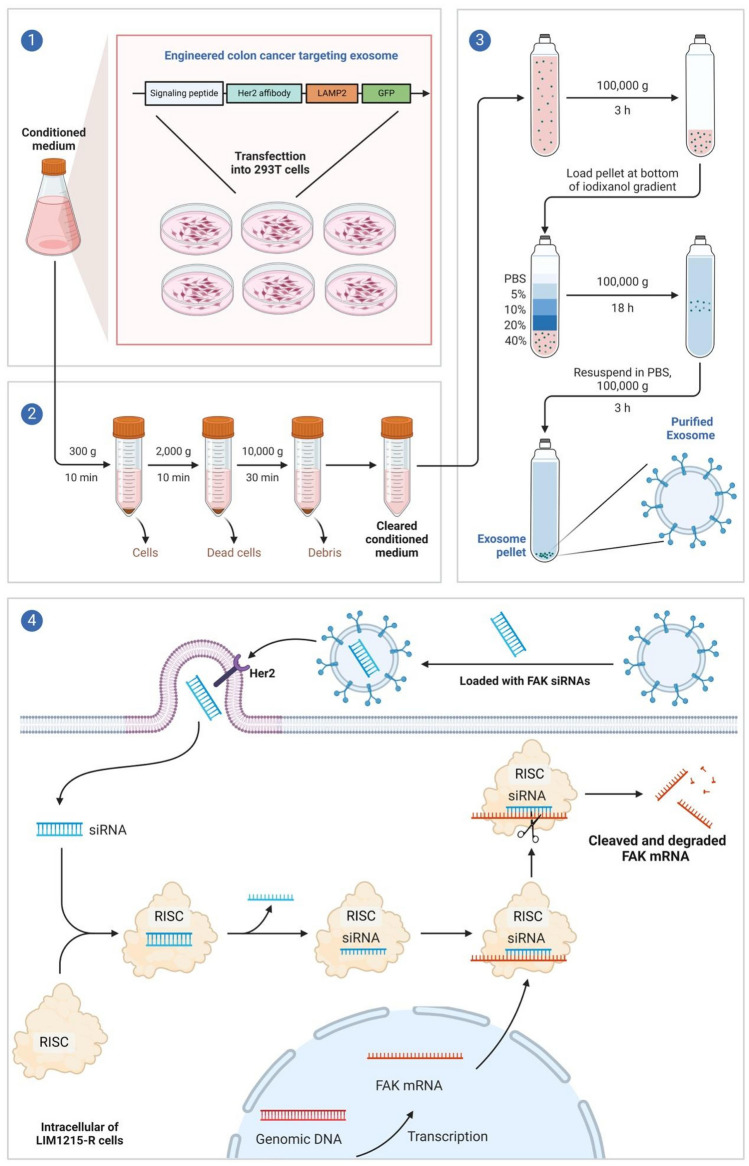
Schematic diagram shows the engineered exosomes targeted delivery of FAK siRNA inhibits cetuximab resistance via activating paraptosis in colon cancer

siRNA holds significant potential in treating CRC by silencing oncogenic and multidrug resistance genes, but its application is hindered by issues such as poor stability [46]. Engineered exosomes refer to a class of modified exosomes processed through biotechnological methods, exhibiting enhanced drug-carrying efficiency, targeting specificity, and resistance to clearance by the organism, often showing superior ability in delivering RNA inhibitors and inhibiting tumor growth compared to liposomes [47]. Lamp2, enriched on the exosomal membrane, and HER-2, highly expressed in CRC. Based on this, it has been validated that the fusion proteins of Her2 and LAMP2 expressed on the exosomal membrane significantly improve exosome targeting to cancer cells. Multiple administrations of these exosomes have shown no acute toxicity to the mouse hematopoietic system and major organs [48]. In this study, we employed this strategy to construct CT-Exo-siFAK1 (Fig. 9). According to our research, LIM1215 and LIM1215-R cells effectively absorb these engineered exosomes loaded with FAK siRNA, which activates paraptosis and inhibits cetuximab-resistant cells growing in vitro. Moreover, CT-Exo-siFAK1 demonstrates the ability to suppress the proliferation and metastasis of resistant colon cancer cells in vivo. In the subcutaneous transplant tumor model of resistant mice treated with CT-Exo-siFAK1, there is a reduction in tumor volume and weight, accompanied by an elevation in paraptosis levels. In the hepatic metastasis model of resistant mice, CT-Exo-siFAK1 treatment decreases the number and size of liver metastatic lesions, concomitant with activation of paraptosis. These results suggest that the constructed CT-Exo-siFAK1 can effectively activate paraptosis and enhance the sensitivity of resistant cells to cetuximab.

In this investigation, we elucidated the involvement of paraptosis in the emergence of cetuximab resistance which is one of the mechanisms contributing to resistance against cetuximab treatment in CRC. Knocking down FAK can activate paraptosis in CRC cells to reverse resistance to cetuximab. FAK serves as one of the target choices influencing the therapeutic efficacy of cetuximab through the regulation of paraptosis mechanisms. In our conducted loading experiments, it has been confirmed that engineered exosome delivery of FAK siRNA can effectively reverse resistance to cetuximab by activating paraptosis in CRC. These findings unveil the critical roles of FAK and paraptosis in CRC resistance to cetuximab, providing a promising strategy for overcoming cetuximab resistance.

Nevertheless, there are certain limitations to this study. Firstly, the design of paraptosis-related indicators detection in this study is relatively simple and could be further improved by integrating other cell death pathways for a more comprehensive evaluation system. Secondly, further confirmation of our conclusions awaits the collection of clinical samples from patients undergoing cetuximab treatment in future studies. Additionally, the study utilized xenografts derived from cell lines in Balb/c female nude mice, and the use of patient-derived xenograft (PDX) models from cetuximab-resistant CRC patients would contribute further to understanding the role of paraptosis and FAK in cetuximab resistance in CRC.

## Supplementary Information

Below is the link to the electronic supplementary material.

Supplementary file1 (DOCX 22.8 KB)

Supplementary file2 (JPG 790 KB)

## Data Availability

All the data and materials supporting the conclusion of this study have been included in the article and the supplemental data.
