# Supplementary material for: Targeted delivery of FAK siRNA by engineered exosomes to reverse cetuximab resistance via activating paraptosis in colon cancer
Source: Apoptosis. 2024 Jul 3;29(11-12):1959–77. doi: 10.1007/s10495-024-01986-x (PMC11550291; doi:10.1007/s10495-024-01986-x)
Supplement: Supplementary file 1 — Supplementary file1 (DOCX 22.8 KB) [file 10495_2024_1986_MOESM1_ESM.docx]

# Supplementary Tables

**Supplementary table 1. Primers for RT-qPCR**

| **Gene name** | **Forward primer (5’-3’)** | **Reverse primer (5’-3’)** |
| --- | --- | --- |
| ATF3 | ACTGTCAGCGACAGACCCCT | TCCCATTCTGAGCCCGGACA |
| CHOP | AGGAGGAGCCAGAACCAGCA | TACTTCCCTGGTCAGGCGCT |
| HERP | ACCTGCATCACGTTGGGTGG | TCTGGAGGGAGGTGGTTGGG |
| TRIB3 | CAGCCGATGTCTGGAGCCTG | TGGGATCGGGTTGGGGCTAA |
| CAV1 | GCGACCCTAAACACCTCAAC | ATGCCGTCAAAACTGTGTGTC |
| PAK3 | CCAGGCTTCGCTCTATCTTCC | TCAAACCCCACATGAATCGTATG |
| PAK6 | ACCAATAGGCATGGAATGAAGG | GCGGTCGGAAAGAGGAGTTG |
| PDGFA | GCAAGACCAGGACGGTCATTT | GGCACTTGACACTGCTCGT |
| PDGFB | CTCGATCCGCTCCTTTGATGA | CGTTGGTGCGGTCTATGAG |
| PDGFD | TTGTACCGAAGAGATGAGACCA | GCTGTATCCGTGTATTCTCCTGA |
| PIK3R3 | TACAATACGGTGTGGAGTATGGA | TCATTGGCTTAGGTGGCTTTG |
| GAPDH | GCGGGGCTCTCCAGAACATC | TCCACCACTGACACGTTGGC |

**Supplementary Table 2.** **Antibodies for Western Blot and IF**

| **Gene name** | **Cat. Log** | **Company** |
| --- | --- | --- |
| ATF-3 | 18665 | Cell Signaling Technology |
| IRE1α | 3294 | Cell Signaling Technology |
| IRE1 (phospho S724) | Ab48187 | Abcam |
| CHOP | 2895 | Cell Signaling Technology |
| Goat Anti-Rabbit-HRP | 7074 | Cell Signaling Technology |
| HRP-GAPDH | 8884 | Cell Signaling Technology |
| GRP78 | GB12098 | Servicebio |
| FAK | 3285 | Cell Signaling Technology |
| Phospho-FAK (Tyr397) | 3283 | Cell Signaling Technology |
| CAV1 | 3267 | Cell Signaling Technology |
| PAK3 | 2609 | Cell Signaling Technology |
| PIK3R3 | 27035-1-AP | Proteintech |
