# Supplementary figures and images for: Targeted delivery of FAK siRNA by engineered exosomes to reverse cetuximab resistance via activating paraptosis in colon cancer
Source: Apoptosis. 2024 Jul 3;29(11-12):1959–77. doi: 10.1007/s10495-024-01986-x (PMC11550291; doi:10.1007/s10495-024-01986-x)

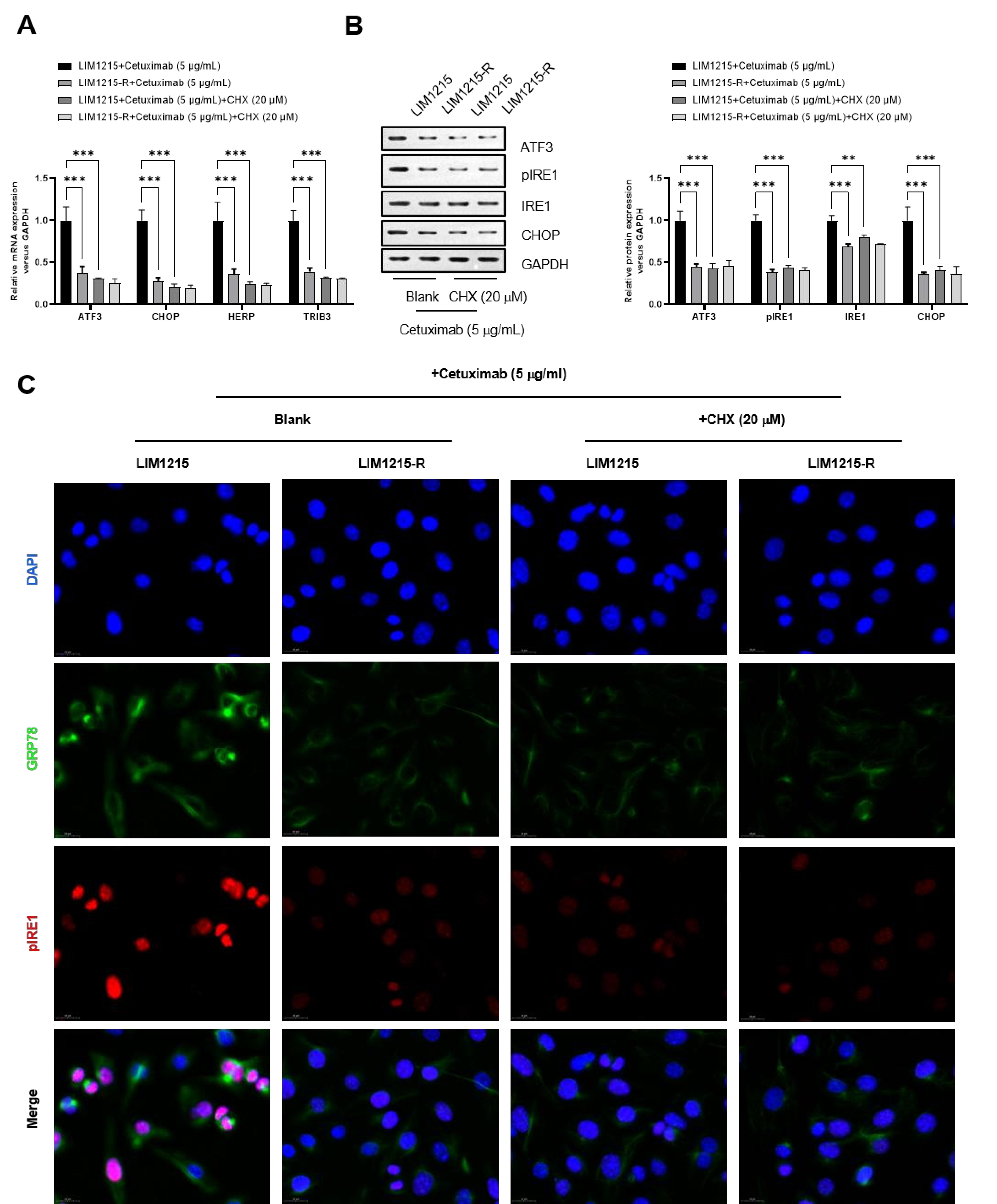

Supplement: Supplementary file 2 — Supplementary file2 (JPG 790 KB) [file 10495_2024_1986_MOESM2_ESM.jpg]
